# Time-resolved IR and X-ray spectroscopy as complementary electronic structure probes of photoinduced ligand-exchange in molybdenum hexacarbonyl

**DOI:** 10.1039/d6cc01470d

**Published:** 2026-03-30

**Authors:** Raphael M. Jay, Timo Dederichs, Marc-Oliver Winghart, Debkumar Rana, Ru-Pan Wang, Torsten Leitner, Robert Stefanuik, Camelia N. Borca, Grigory Smolentsev, Ambar Banerjee, Michael Odelius, Nils Huse, Thomas Huthwelker, Erik T. J. Nibbering, Philippe Wernet

**Affiliations:** a Department of Physics and Astronomy, Uppsala University 75120 Uppsala Sweden raphael.jay@physics.uu.se philippe.wernet@physics.uu.se; b Max Born Institute for Nonlinear Optics and Short Pulse Spectroscopy 12489 Berlin Germany erik.nibbering@mbi-berlin.de; c Department of Physics and Center for Free-Electron Laser Science, University of Hamburg 22761 Hamburg Germany; d Paul Scherer Institute CH-5232 Villigen PSI Switzerland; e Research Institute for Sustainable Energy (RISE), TCG Centres for Research and Education in Science and Technology (TCG-CREST) Kolkata 700091 India; f Department of Physics, Stockholm University, AlbaNova University Center 106 91 Stockholm Sweden

## Abstract

Photoinduced ligand-exchange dynamics in molybdenum hexacarbonyl have been studied using time-resolved IR and X-ray spectroscopy. We find that the energies of the unoccupied molybdenum 4d-derived orbitals that are accessed through X-ray transitions correlate with red-shifts in CO stretching modes. This provides complementary electronic structure information from the metal and the ligand perspective.

## Abstract 2

Time-resolved X-ray and infrared spectroscopy reveal electronic structure changes from the metal and ligand perspect following ligand-exchange in molybdenum hexacarbonyl.

## Graphical abstract

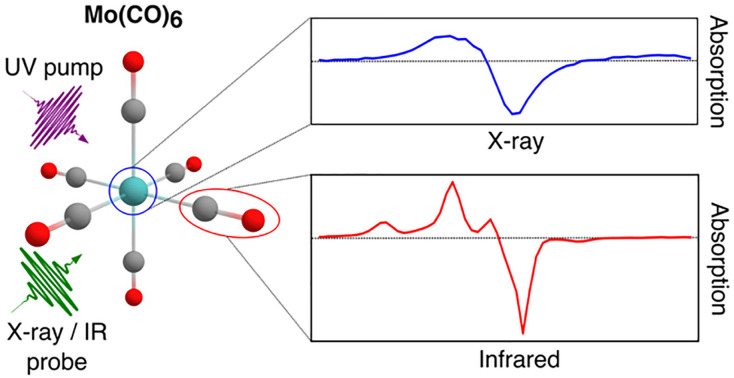

Photoinduced ligand-exchange plays a pivotal role as the starting point of a wide range of photocatalytic reactions.^1^ To spectroscopically study the underlying ligand dissociation and association dynamics, transition metal carbonyls have long served as popular model systems.^2,3^ By leveraging the bright IR-active CO stretching marker modes, time-resolved IR spectroscopy has been particularly successful in studies of metal carbonyls. The method has been key in identifying intermediates and measuring timescales in a wide range of ligand-exchange reactions from classic model systems such as iron pentacarbonyl and group 6 hexacarbonyls^4–7^ to transition metal carbonyl catalysts used in photochemical C–H bond activation.^8–11^

In recent years, time-resolved X-ray absorption spectroscopy (XAS) has emerged as a complementary probe of photo-induced ligand-exchange dynamics.^12–16^ At metal L-edges, the underlying strong dipole-allowed metal 2p → nd transitions provide unique access to the electronic structure locally around the metal center.^17–19^ In selected cases, the interpretation of the observed X-ray spectroscopic signatures has been found to be fully consistent with previously observed shifts in the ligand marker modes and their interpretation in terms of changing metal–ligand interactions.^15,20,21^ However, a direct and comprehensive comparison of X-ray and IR spectroscopic fingerprints has thus far not been attempted.

Here, we use time-resolved IR spectroscopy and time-resolved XAS at the metal L-edge to systematically establish the complementarity of the two methods in terms of their sensitivity to changes in the valence electronic structure. Specifically, we follow the photoinduced ligand-exchange dynamics in molybdenum hexacarbonyl (Mo(CO)_6_) in 1-pentanol solution. It has been established that UV excitation of Mo(CO)_6_ leads to ultrafast dissociation of a CO ligand followed by the rapid association of a 1-pentanol solvent molecule (see photochemical pathway shown in Scheme 1).^22,23^ Here, we now observe with time-resolved IR and X-ray absorption spectroscopy in the tender range,^15,17,19,24^ how metal–ligand covalency changes upon CO to 1-pentanol ligand-exchange in Mo(CO)_6_ as well as the ensuing interconversion of CH to OH-coordinated reaction products from Mo(CO)_5_-R-OH to Mo(CO)_5_-OH-R.

**Scheme 1:**
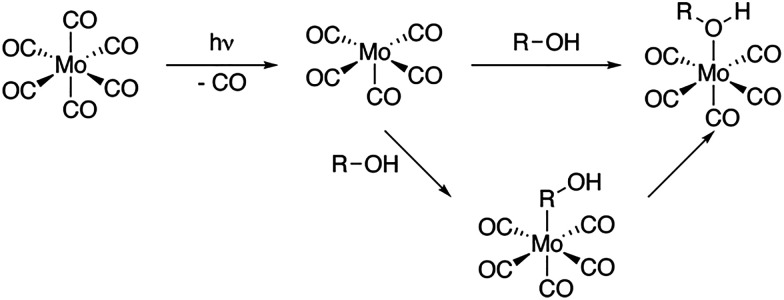
Pathways of photoinduced ligand-exchange in Mo(CO)_6_ in an alcohol solution.

Fig. 1 shows our time-resolved IR data of Mo(CO)_6_ in 1-pentanol photoexcited at 266 nm (see SI for experimental details). The transient difference spectra in Fig. 1a show a strong depletion of the CO stretching bands of the Mo(CO)_6_ parent complex at 1985 cm^−1^ concomitant with the emergence of transient absorption in the range of 1870 cm^−1^ to 1975 cm^−1^. The transient absorption bands at 1895 cm^−1^ and 1940 cm^−1^ fully develop over several 100 ps. Since these bands are absent in our measurements of Mo(CO)_6_ in pentane (see Supplementary Information), we assign them to CO stretching vibrations of the final Mo(CO)_5_-OH-R species, analogously to previous time-resolved IR measurements on Cr(CO)_6_.^5^

**Fig. 1:**
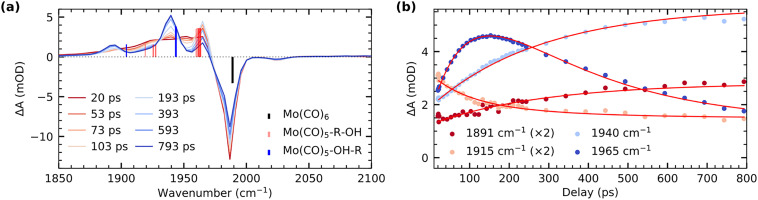
(a) Transient difference IR spectra of Mo(CO)_6_ in 1-pentanol measured at different pump–probe delays following photoexcitation. Calculated energies and relative intensities of CO stretching frequencies of Mo(CO)_6_, Mo(CO)_5_-OH-R and the 5 possible Mo(CO)_5_-R-OH species are shown as vertical lines. (b) Delay traces measured at different wavenumbers and fitted with a biexponential function yielding time constants of (94 ± 9) ps and (280 ± 20 ps) ps.

Transient intensity in the range of 1910 cm^−1^ to 1930 cm^−1^ as well as at 1965 cm^−1^ can instead be assigned to intermediate Mo(CO)_5_-R-OH species.^7^ In the 1-pentanol solution addressed here, we hence observe Mo(CO)_5_-R-OH to Mo(CO)_5_-OH-R interconversion on timescales of several 100 ps. For comparison, in methanol solution, where an extended carbon chain is absent, the Mo(CO)_5_-OH-R product bands directly appear within the vibrational cooling timescales of about 10 ps^7^ (see SI). The timescale of interconversion from CH to OH-coordinated products can be deduced from the delay traces shown in Fig. 1b. The data is fitted globally with a biexponential function. A fast time constant of 94 ± 9 ps is assigned to the decay of CO stretching excitations, which have previously been reported to relax independently of the initial vibrational energy redistribution in hot ligand-exchanged photoproducts as well as energy dissipation into the solvent.^7,14^ In alkanes, relaxation timescales of 160 ps have been reported for the decay of these CO stretching excitations.^7^ In alcohols, hydrogen bonding leads to a more anharmonic CO stretching potential, which enhances coupling to other modes and rationalizes the shorter timescale observed here. The second time constant of 280 ± 20 ps, reflected in the decay of the transient absorption at 1965 cm^−1^ with concomitant rise of absorption bands at 1895 cm^−1^ and 1940 cm^−1^, is assigned to the interconversion of CH to OH-bound species. Mo(CO)_5_-R-OH to Mo(CO)_5_-OH-R migration observed here is faster than Cr(CO)_5_-R-OH to Cr(CO)_5_-OH-R interconversion reported earlier.^5^ This difference may be related to the larger spatial extent and thus higher covalency of Mo compared to Cr.^25^ The resulting stronger metal-OH bonds in Mo compared to Cr may then induce a pull-effect that accelerates the migration towards the final OH-bound photoproduct. Our assignments of transient absorption bands are supported by our calculations of CO stretching frequencies shown in Fig. 1a. Besides some small relative shifts between calculated and experimental vibrational frequencies, the transient absorption bands at 1895 cm^−1^ and 1940 cm^−1^ can be assigned to CO stretching vibrations of the final Mo(CO)_5_-OH-R product. Transient intensity in the range of 1910 cm^−1^ to 1930 cm^−1^ as well as at 1965 cm^−1^ can instead be clearly attributed to the CO stretching vibrations of the intermediate Mo(CO)_5_-R-OH species. Overall, we hence find that CO to R-OH and OH-R ligand-exchange splits and red-shifts the quasi-degenerate CO stretching bands in the Mo(CO)_5_-R-OH and Mo(CO)_5_-OH-R species with respect to the Mo(CO)_6_ parent complex.

Our time-resolved XAS data of Mo(CO)_6_ in 1-pentanol photoexcited at 266 nm is shown in Fig. 2 (see SI for experimental details). Upon ligand-exchange, strong depletion of the Mo(CO)_6_ main edge at 2525 eV can be observed concomitant with the emergence of a transient pre-edge centered at 2522.5 eV. At the earlier measured time delay of 0.3 ns, at which the IR data suggests that a mix of Mo(CO)_5_-R-OH and Mo(CO)_5_-OH-R species is present, the pre-edge is slightly higher in intensity than at later timescales (>160 ns), when all photo-products have interconverted into the final OH-coordinated Mo(CO)_5_-OH-R product.^26,27^ A delay trace measured at 2521.4 eV in the rising edge of the transient pre-edge is shown in the inset in Fig. 2a. While the signal-to-noise ratio of the delay trace does not allow for a robust extraction of a time constant, the data is consistent with the 280 ps timescale of interconversion extracted from the IR data (accordingly, a fit with a fixed time constant of 280 ps agrees well the XAS data).

**Fig. 2:**
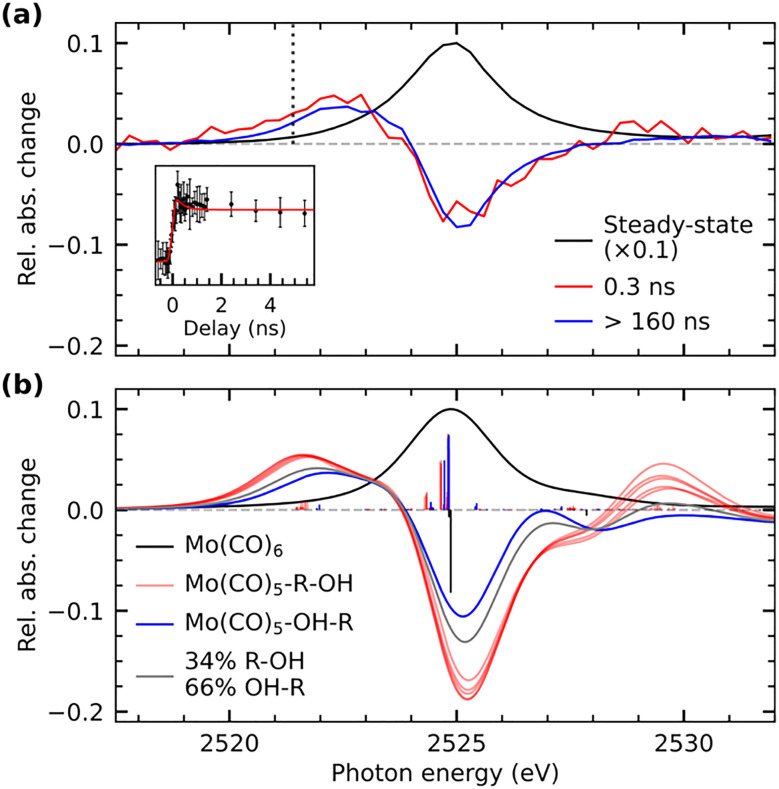
(a) Steady-state Mo L_3_-edge absorption spectrum of Mo(CO)_6_ in 1-pentanol and transient difference spectra measured at different pump–probe delays. Difference intensities are displayed relative to the maximum of the steady-state absorption spectrum. The inset shows a delay trace measured at the onset of the transient pre-edge at 2521.4 eV. To guide the eye, a fit with a fixed time constant of 280 ps is additionally shown. (b) Calculated L-edge absorption spectrum of Mo(CO)_6_ (scaled to the measured spectrum of Mo(CO)_6_) as well as calculated difference spectra of the Mo(CO)_5_-OH-R and the 5 possible Mo(CO)_5_-R-OH species with respect to the Mo(CO)_6_ parent complex (single X-ray transitions are displayed as sticks, the Mo(CO)_5_-OH-R difference spectrum is scaled to match the pre-edge intensity of the experimental difference spectrum at delays >160 ns and the Mo(CO)_5_-R-OH spectra are scaled accordingly). A spectrum representing a mix of Mo(CO)_5_-R-OH and Mo(CO)_5_-OH-R contributions is shown additionally.

The experimentally observed emergence of a transient pre-edge XAS peak and its varying intensity depending on CH or OH coordination is reproduced by our calculations shown in Fig. 2b (see SI for computational details). The theoretical overestimation of transient intensities in the pre-edge and the main-edge depletion in the spectrum at 0.3 ns are due to the fact that at this timescale a majority of species (∼66% based on the time-resolved IR data) have already interconverted to the OH-coordinated configuration. To simulate the expected spectral behavior at 0.3 ns delay, an additional calculated difference spectrum is shown in Fig. 2b, which is a mixture of Mo(CO)_5_-R-OH and Mo(CO)_5_-OH-R species and reproduces the measured difference spectrum at 0.3 ns well. This good agreement between experiment and theory therefore allows for a robust assignment and characterization of the underlying transitions.

In agreement with our earlier analysis of the L-edge absorption spectrum of Cr(CO)_6_,^14^ the main absorption line in Mo(CO)_6_ at 2525 eV is dominated by substantially mixed transitions of Mo 2p electrons into unoccupied Mo 4d(e_g_)-derived orbitals as well as into unoccupied CO π*-derived orbitals of t_2g_ symmetry^28^ (see Supplementary Information for plots of molecular orbitals). The latter adopt substantial 4d character through backdonation from the occupied Mo 4d orbitals with the same symmetry. Upon ligand-exchange and breaking of *O*_h_ symmetry, the manifold of CO π* orbitals splits. This splitting is reflected by a broader distribution of single X-ray transitions in the Mo L_3_ main edge absorption in the CH and OH-coordinated species compared to the Mo(CO)_6_ parent complex (see sticks in Fig. 2b). Importantly, upon removal of a CO ligand, one CO π* orbital originating from *t*_1u_ manifold in *O*_h_ symmetry is substantially stabilized and acquires substantial Mo d character, an effect observed before,^14,29^ and consistent with fundamental notions of metal–ligand orbital interactions.^30^ Analogous to previous XAS-based observations in the ligand-exchange dynamics of Cr(CO)_6_ and W(CO)_6_,^6,14^ this orbital mixing enables Mo 2p excitations into this CO π*-hybridized 4d orbital (see Supplementary Information for plots of molecular orbitals) and leads to marked transient pre-edge intensity in the Mo L-edge absorption spectrum at 2520–2523 eV.

To deduce a comprehensive picture of the electronic structure changes in Mo(CO)_6_ upon ligand exchange from both the Mo and the ligand perspectives with an emphasis on changes in bond covalency, we turn to the fragment charge decomposition analysis shown in Table 1. This analysis provides calculated values of the total electronic charge transfer between the (CO)_5_ fragment and the Mo-L fragments (where L = CO, R-OH or OH-R) as well as between the Mo(CO)_5_ and the L fragments. A schematic summary of the associated changes in covalency upon photo-induced ligand-exchange is shown in Scheme 2. Exchange of the strongly π-accepting CO ligand by a weakly π-accepting alcohol (in both CH and OH-coordinated geometries) leads to an increase in π-backdonation onto the remaining CO ligands (see in Table 1 the increasing charge transfer from Mo-L to (CO)_5_, when exchanging CO for R-OH and OH-R). This increase in backdonation compared to CO is more pronounced for OH-R than R-OH due to how *σ*-donation by OH-R and R-OH ligands varies compared to CO. While CO is the strongest *σ*-donor, net charge transfer onto Mo(CO)_5_ is minimal (see Table 1) because donation and backdonation act in different directions and are balanced in magnitude. For the (almost) exclusively *σ*-donating R-OH and OH-R ligands, effective charge transfer onto the Mo(CO)_5_ fragment is found (Table 1). This charge transfer is larger in Mo(CO)_5_-OH-R compared to Mo(CO)_5_-R-OH because the OH-R ligand is a stronger *σ*-donor than the R-OH one. The larger charge transfer from the OH-R ligand to Mo(CO)_5_ compared to the R-OH and CO ligands, in turn, enables the increased Mo-OH-R to (CO)_5_ backdonation compared to Mo(CO)_5_-R-OH and Mo(CO)_6_.

**Table 1:** Fragment charge decomposition analysis of the three Mo(CO)_5_-L complexes (L = CO, R-OH, OH-R) at the B3LYP level of theory. Calculated electronic charge transfers for Mo(CO)_5_-R-OH are presented as the average of the 5 possible species

Species	Mo-L → (CO)_5_	L → Mo(CO)_5_
Mo(CO)_6_	0.19	−0.04
Mo(CO)_5_-R-OH	0.37	0.11
Mo(CO)_5_-OH-R	0.53	0.15

**Scheme 2:**
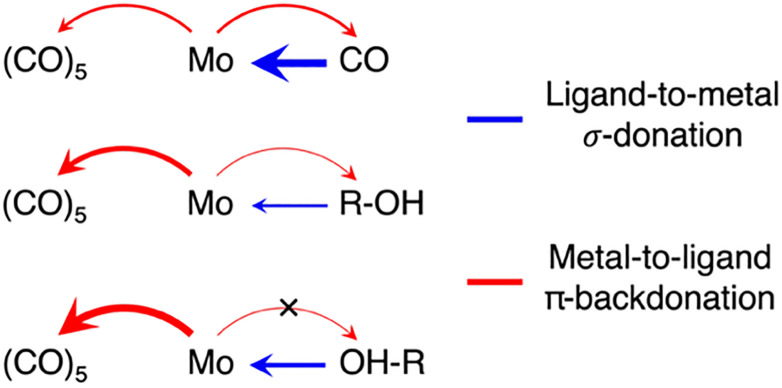
Schematic depiction of donation and backdonation interactions in the three Mo(CO)_5_-L species (L = CO, R-OH, OH-R).

This theoretical prediction of unbalanced donation and backdonation in Mo(CO)_5_-CH and Mo(CO)_5_-OH compared to Mo(CO)_6_ is consistent with the observed spectral trends in our time-resolved IR and X-ray absorption data. Within the framework of the Tolman electronic parameter,^31^ an increase in metal-to-CO π-backdonation along the series from Mo(CO)_6_ to Mo(CO)_5_-R-OH and Mo(CO)_5_-OH-R (see Scheme 2) increasingly weakens the CO bonds consistent with the observed increasingly red-shifted CO stretching bands (see Fig. 1a). The concomitant decrease in ligand-to-metal *σ*-donation when exchanging the strong *σ*-donor CO by an alcohol (see Scheme 2) is reflected in the appearance of the low-energy pre-edge peak in the Mo L-edge absorption spectra of Mo(CO)_5_-R-OH and Mo(CO)_5_-OH-R compared to Mo(CO)_6_ (see Fig. 2). This pre-edge peak is due to excitations of Mo 2p electrons into the anti-bonding combination of the Mo 4d(e_g_)-derived molecular orbital representing the metal–ligand *σ*-donation interaction. For R-OH and OH-R ligands and compared to CO, *σ*-donation is smaller, the anti-bonding 4d(e_g_)-derived orbital is stabilized (its energy is smaller compared to CO as a ligand) and excitations of Mo 2p electrons into this orbital appear as a pre-edge peak (in Mo(CO)_6_, the strong ligand *σ*-donation pushes the same transitions up in energy and into the main edge of the Mo L-edge spectrum). Upon R-OH to OH-R exchange, the increase in *σ*-donation (see Scheme 2) destabilizes the same orbital slightly, which is evident in the minor shift of the pre-edge intensity to higher incidence energy (see Fig. 2).

Besides its sensitivity to the degree of *σ*-donation, L-edge absorption additionally has been shown to exhibit sensitivity to backdonation interactions.^18,32^ In octahedral systems, these interactions are typically evaluated *via* the degree of hybridization between occupied metal 4d(t_2g_) orbitals and the unoccupied ligand π* orbitals of the same symmetry. Here, however, the close energetic proximity of the CO π* t_2g_ manifold to the unoccupied 4d(e_g_)-derived orbitals leads to highly mixed transitions in the main edge for all three species Mo(CO)_6_, Mo(CO)_5_-R-OH and Mo(CO)_5_-OH-R that are consequently largely uninformative regarding the degree of π-backdonation (see transitions with strongest oscillator strength at 2524.8 eV in Fig. 2b). Instead of being sensitive to π-backdonation onto the (CO)_5_ fragment, the L-edge exhibits sensitivity to the minor backdonation from the Mo center onto the coordinated CH group in Mo(CO)_5_-R-OH species. This effect can be observed *via* the calculated transitions at 2529 eV (Fig. 2b), which correspond to Mo 2p excitations into the σ* orbital of the C-H group that has adapted minor Mo 4d character *via* backdonation from the metal.^33,34^ At a time delay of 0.3 ns, when Mo(CO)_5_-R-OH species are present, transient intensity is indeed experimentally observable at the same energy, albeit at limited signal-to-noise ratio.

In summary, we have used time-resolved IR and X-ray spectroscopy to study the photoinduced ligand-exchange dynamics in Mo(CO)_6_ in 1-pentanol solution. We show how CO specific IR transitions provide high sensitivity to π-backdonation from Mo to the carbonyl manifold, whereas the Mo-specific X-ray absorption spectra predominantly reflect changes in ligand-to-Mo *σ*-donation for the different intermediates with different ligands along the photochemical pathway. This complementary information content is not restricted to ligand-exchange dynamics, but enables detailed insight into the evolution of the valence electronic structure from the metal and the ligand perspective in all photocatalytic applications involving transition metal complexes and catalysts with ligands that provide strong IR marker modes.

R. M. J., E. T. J. N. and P. W. originated the project concept. R. M. J., T. D., M.-O. W., D. R., R.-P. W., T. L., R. S., C. N. B., G. S., N. H., T. H., E. T. J. N. and P. W. planned and executed the experiments. R. M. J and T. D. analyzed the experimental data. R. M. J, T. D., A. B. and M. O. performed and analyzed the theoretical calculations. R. M. J. and P. W. wrote the paper with input from all the authors.

## Conflicts of interest

There are no conflicts to declare.

## Supplementary Material

CC-062-D6CC01470D-s001

## Data Availability

Additional data are available from the authors upon request. All data supporting the conclusions of this article are included in the main text and the supplementary information (SI). Supplementary information is available. See DOI: https://doi.org/10.1039/d6cc01470d.
