# Supplementary material for: Time-resolved IR and X-ray spectroscopy as complementary electronic structure probes of photoinduced ligand-exchange in molybdenum hexacarbonyl
Source: Chem Commun (Camb). 2026 Mar 30;62(31):8051–5. doi: 10.1039/d6cc01470d (PMC13058699; doi:10.1039/d6cc01470d)
Supplement: CC-062-D6CC01470D-s001 [file CC-062-D6CC01470D-s001.pdf]

Supplementary Information (SI) for ChemComm.
This journal is © The Royal Society of Chemistry 2026

Supporting Information for

Time-resolved IR and X-ray spectroscopy as complementary electronic structure probes of photoinduced ligand-exchange in molybdenum hexacarbonyl

Raphael M. Jay,* Timo Dederichs, Marc-Oliver Winghart, Debkumar Rana, Ru-Pan Wang, Torsten Leitner, Robert Stefanuik, Camelia N. Borca, Grigory Smolentsev, Ambar Banerjee, Michael Odelius, Nils Huse, Thomas Huthwelker, Erik T. J. Nibbering* and Philippe Wernet*

Email: raphael.jay@physics.uu.se, erik.nibbering@mbi-berlin.de, philippe.wernet@physics.uu.se

This PDF file includes:

Experimental details

Computational details

Time-resolved IR data of $Mo(CO)_6$ in pentane and in methanol

Comparison between experimental and calculated X-ray absorption difference spectra

Charge density difference plots

Plots of molecular orbitals

Optimized geometries

Experimental details

The time-resolved X-ray absorption spectroscopy (XAS) measurements were performed at the PHOENIX (PHotons for the Exploration of Nature by Imaging and XAFS) beamline of the Swiss Light Source (SLS, Paul Scherrer Institut, Switzerland) as previously described[1,2]. The molybdenum hexacarbonyl ($Mo(CO)_6$) sample (15 mM in 1-pentanol, purchased from Sigma-Aldrich) was injected into the experimental chamber using a 50 µm cylindrical jet at flow rates of around 1 mL/min. This ensured that the sample was fully replenished between consecutive X-ray and laser pulses. The sample was collected by a catcher system below the interaction zone and transported out of the experimental chamber. To avoid sample degradation, the sample was not recirculated. An avalanche photodiode (APD) was used to measure the total fluorescence yield (TFY) as a function of the incident photon energy in the range of the Mo L_3-edge. X-ray absorption spectra were then generated by normalizing the measured signal to the I_0 intensity of the incoming monochromatized X-ray beam determined using another APD diode, which measures the scattering from a mylar foil. The experimental chamber was kept under a 500 mbar He atmosphere to reduce X-ray scattering noise and increase X-ray transmission. The fourth harmonic of a 1065 nm Neodymium-doped yttrium orthovanadate ($Nd:YVO_4$) amplified laser system at a fluence of ~130 mJ/cm^2 was used to optically excite the sample. The laser pulse duration was approximately 10 ps and the laser repetition rate was 130 kHz. The time-resolution of the experiment was estimated by the rise-time of the delay trace measured at 2521.4 eV and amounted to ~250 ps, which is predominately determined by the X-ray pulse length. Time-resolved XAS measurements in the delay range <100 ns were carried out using consecutive laser-on and laser-off X-ray pulses from the Camshaft bunch of the SLS fill pattern. X-rays originating from the multibunch of the SLS fill pattern were instead used and integrated over the range of several 100 ns for spectrum measurements in the delay region beyond 100 ns[1].

Femtosecond UV-pump/IR-probe experiments on 10 mM samples of $Mo(CO)_6$ in 1-pentanol, pentane and methanol were carried out on an experimental setup described previously[3]. One fraction of the output from a titanium sapphire (Ti:Sa) amplifier system (800 nm, 35 fs, 1kHz, Solstice Ace, Spectra Physics) was used to generate UV-pump pulses via third harmonic generation (266 nm, 0.65 µJ), while the other part was sent to a home-built double-pass optical parametric amplifier (OPA) followed by difference frequency generation from signal and idler to generate tuneable mid-IR probe pulses. The polarization between the two pulses was set to the magic angle (54.7°). Both pulses were focused onto a liquid flow cell filled with the sample solutions, using a UV pump spot size of 280 µm diameter. The typical experimental temporal resolution is ~350 fs, resulting mainly from the group velocity mismatch of the UV and mid-IR beams. Transmitted mid-IR probe light intensities are recorded with a Mercury cadmium telluride (HgCdTe) detector (Infrared Associates MCT-6400) and normalized by a fraction of the mid-IR beam that propagates through an unpumped region of the sample. Finally, transient mid-IR difference absorption spectra are obtained by chopping the UV pump pulses at 500 Hz, which results in alternating pumped and unpumped spectra being recorded.

Computational details

Quantum chemical calculations were performed with the ORCA software package[4]. All calculations used the RIJCOSX[5] protocol for computational efficiency and a polarizable continuum model[6] to account for effects of the 1-pentanol solvent. Molecular geometries were optimized within the singlet ground state of all species using the TPSSh[7] functional and the def2-TZVPP[8] basis set followed by a calculation of the vibrational frequencies. Mo L_3-edge absorption spectra were calculated using time-dependent density functional theory (TD-DFT) at the B3LYP level of theory[9,10]. Scalar relativistic effects in the TD-DFT calculations were included in the zeroth order regular approximation[11] (ZORA). The SARC-ZORA-TZVPP basis

set[12] was used for the Mo atom and the ZORA-def2-TZVPP basis set for all other atoms. Calculated transitions were convolved with a Voigt-function including a Lorentzian broadening of 1.6 eV FWHM to account for the Mo 2p core-hole lifetime[13] as well as a Gaussian broadening of 1.6 eV FWHM to account for inhomogeneous and conformational broadening. To account for core-hole relaxation effects, a uniform shift of -9.4 eV was applied to all X-ray absorption calculated spectra, which was determined by aligning the experimental and calculated X-ray absorption maxima of the $Mo(CO)_6$ parent complex. The charge decomposition analysis[14] was performed using the Multiwfn program[15] at the same level of theory as the L_3-edge spectrum calculations.

Time-resolved IR data of $Mo(CO)_6$ in pentane

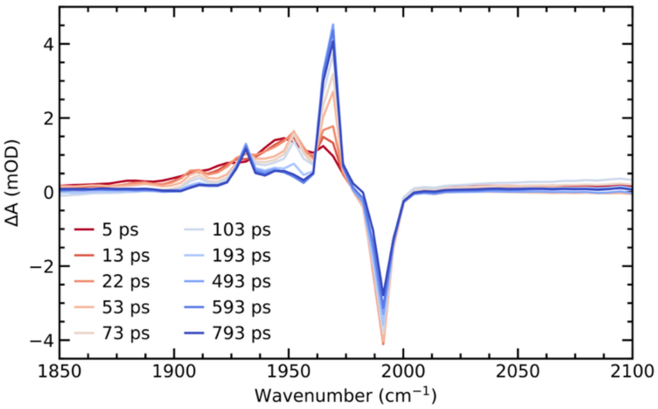

Fig. S1 Transient difference IR spectra of $Mo(CO)_6$ in pentane measured at different pump-probe delays following photoexcitation.

Time-resolved IR data of $Mo(CO)_6$ in methanol

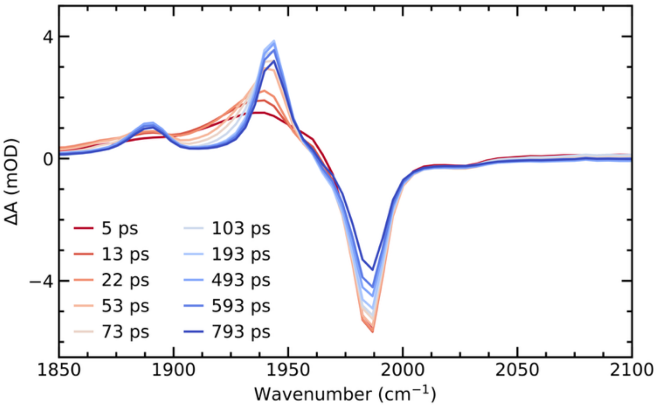

Fig. S2 Transient difference IR spectra of $Mo(CO)_6$ in methanol measured at different pump-probe delays following photoexcitation.

Comparison between experimental and calculated time-resolved XAS spectra

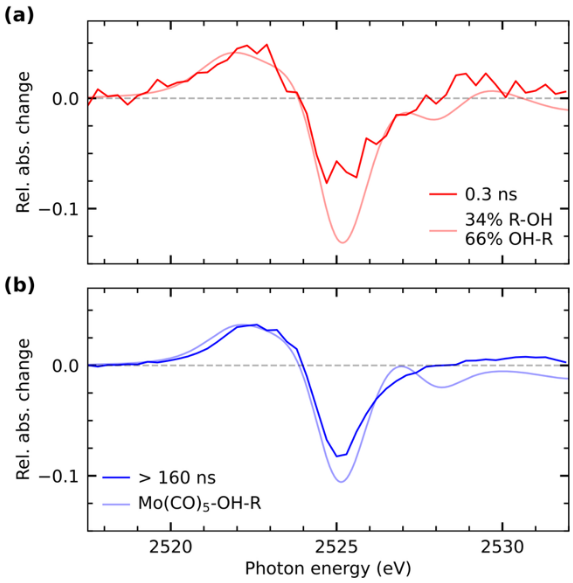

Fig. S3. Direct comparison between **(a)** the measured transient difference XAS spectrum at 0.3 ns and the calculated difference XAS spectrum of a mix of $Mo(CO)_5$-R-OH and $Mo(CO)_5$-OH-R contributions and **(b)** the measured transient difference XAS spectrum for delays > 160 ns and the calculated difference XAS spectrum of the $Mo(CO)_5$-OH-R species. As in the main text, the $Mo(CO)_5$-OH-R difference spectrum is scaled to match the pre-edge intensity of the experimental difference spectrum at delays >160 ns and the mixed spectrum in (a) is scaled accordingly, thereby quantitatively capturing the shape and intensity of the transient pre-edge.

Charge density difference plots

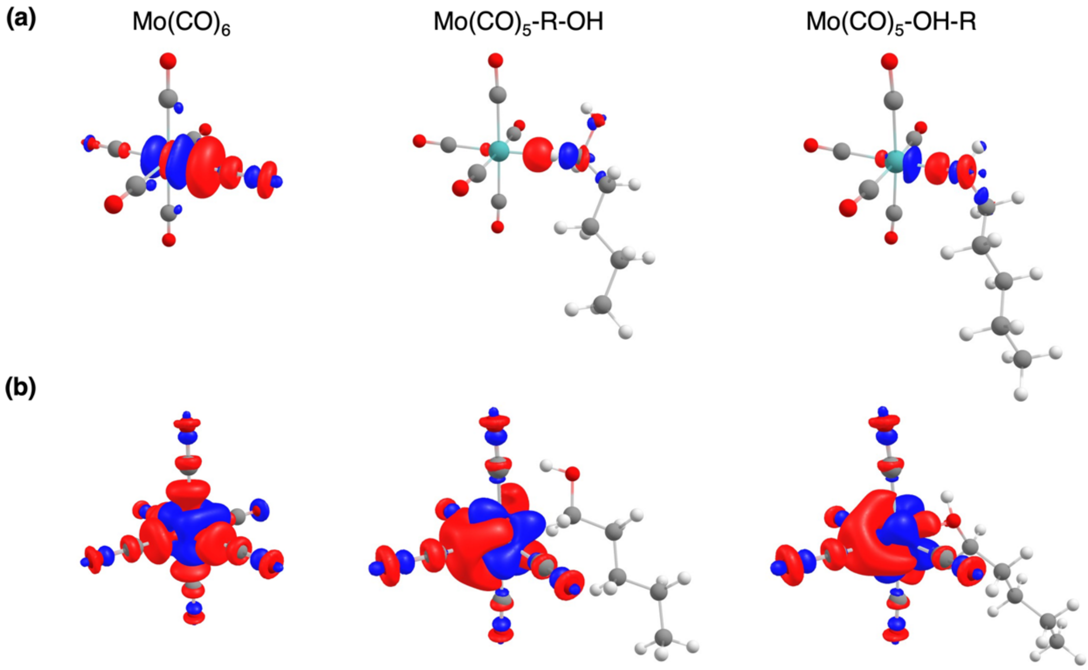

Fig. S4. Charge density difference plots visualizing the fragment charge decomposition analysis for the three considered $Mo(CO)_5$-L species (L = CO, R-OH, OH-R) from the main text. (a) L → $Mo(CO)_5$ (plotted at an isovalue of 0.005) and (b) Mo-L → $(CO)_5$ (plotted at an isovalue of 0.01).

Plots of selected unoccupied orbitals

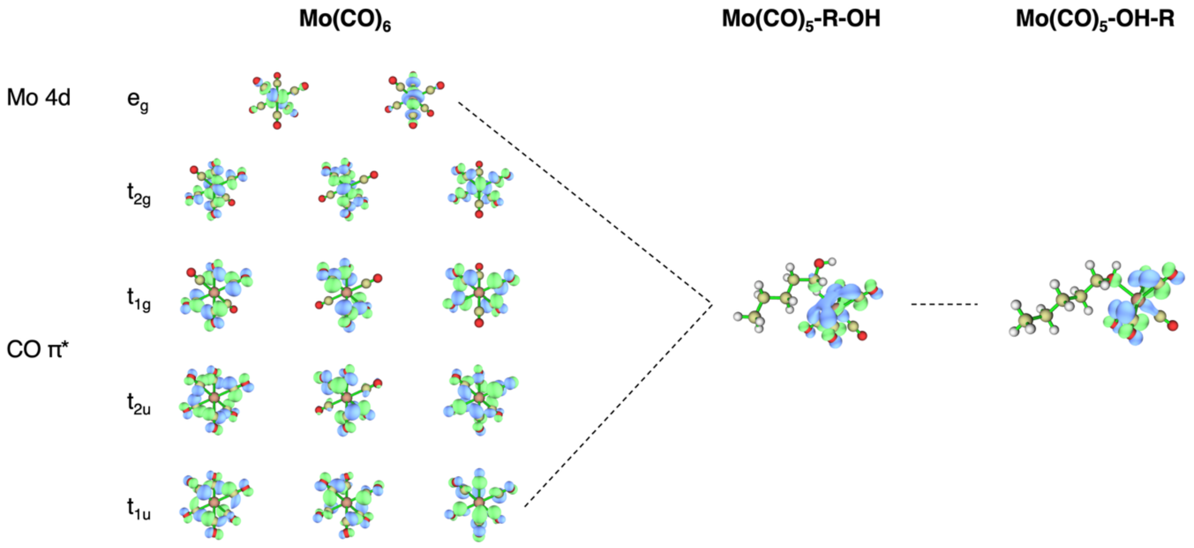

Fig. S5. Selected unoccupied Kohn-Sham orbitals (plotted at an isovalue of 0.05) of the $Mo(CO)_6$ parent complex as well as the ligand-exchanged $Mo(CO)_5$-R-OH and $Mo(CO)_5$-OH-R species.

Optimized geometries (in Å)

$\underline{Mo(CO)_6}$

Mo	-0.75330484455277	0.00000181322289	-1.61123888337775
C	0.91504707789080	-0.91660651798675	-2.40334724897903
C	-0.75065842772641	-1.34355324748136	-0.04861177430428
C	-1.96126126739866	-1.26236911893932	-2.70651179094806
C	-0.75595601680260	1.34355354544140	-3.17386989199008
C	0.45465021991418	1.26237280591898	-0.51596351161373
C	-2.42165640688331	0.91660704383009	-0.81912772119372
O	1.83999803822957	-1.42594784329267	-2.84430544664941
O	-0.74857997984287	-2.09334312250091	0.81584379761096
O	-2.62849623927808	-1.96173885701799	-3.31907636547183
O	-0.75804074722323	2.09333703083957	-4.03833095430417
O	1.12188270853041	1.96174244651242	0.09660375504338
O	-3.34660811485707	1.42594402145367	-0.37816596382222

<u>$Mo(CO)_5$-1-pentanol (OH-bound)</u>

Mo	-0.91807746748924	0.11288569826335	-1.61655000474828
C	0.63369913255364	-0.04702524751706	-2.95517641617507
C	-0.28274823075983	-1.60202632107564	-0.68255191789309
C	-1.99215674926250	-1.02073360548492	-2.80238286766359
C	-1.64872016063027	1.74051970757966	-2.63028090553710
C	-2.53410190480457	0.20227129749528	-0.35788105587363
O	1.45681540555038	-0.18427479022051	-3.74444248051907
O	0.02634702923035	-2.60321224903880	-0.21149676256209
O	-2.63235945817504	-1.69597581151480	-3.49707783837847

O	-2.11138693675473	2.59797897136060	-3.23981073583757
O	-3.47908294914404	0.20644572773985	0.29629876800349
C	1.46777781271879	4.85069351758053	-1.24560463224789
H	1.73886462592953	5.23412064118735	-0.25506688262852
C	2.46223275076327	5.38161946662685	-2.27994490072590
H	3.46108390922739	4.99631192389546	-2.04821408212265
H	2.19850612137556	4.98692319250657	-3.26713343766069
H	0.46932423922446	5.24339383777147	-1.46943717893373
C	2.50111931628467	6.90879341437462	-2.33259822600538
H	1.52041913511229	7.31609466911466	-2.59420250141427
H	2.78759213559900	7.32702347881688	-1.36349406354904
H	3.21996912193714	7.26190928477705	-3.07587214127479
O	0.37105280791177	1.37044744031824	-0.18078461495420
C	0.43763480272204	2.83153116874896	-0.15348208557799
H	-0.56755985549187	3.22300545249585	-0.32606014650494
H	0.76327488770362	3.12060533648667	0.84694561157733
H	0.25527762631071	1.05786209903081	0.72462093442509
C	1.41367611659338	3.32051356652063	-1.20300145632591
H	2.40662360253530	2.91641864844983	-0.98375908460645
H	1.11564296522866	2.93715787171022	-2.18401884628544

Mo(CO)$_5$-1-pentanol (C1H-bound)

Mo	-1.00332087123294	-0.23316045412057	-1.80223479751878
C	0.63785105410938	-1.15175532756786	-2.63175688108211
C	-1.14397469387715	-1.73254832144378	-0.40364552839964
C	-2.13791983993965	-1.28923240627760	-2.99377639456479
C	-0.89194215726006	1.24138835036103	-3.22966559406849
C	-2.70004342111944	0.64364910178145	-1.04540071995189
O	1.51592760866726	-1.69270895253511	-3.13322002891890
O	-1.25223579312817	-2.59546306890741	0.34367485309508
O	-2.80469677287088	-1.92554459701692	-3.69425846884542
O	-0.86069562199249	2.03714446863503	-4.05493983507498
O	-3.68212526764772	1.10039776915979	-0.66701205606371
C	2.28023833692540	2.45348643812401	-1.30271480401834
H	1.59087623592182	3.22205166143155	-1.67025116786352
C	3.70899119424231	3.00149044460346	-1.32839653074820
H	3.78026873079133	3.84784258147012	-0.63661933015688
H	4.39352063073461	2.23430461416976	-0.95064290781411
H	2.20497756850283	1.60751126546849	-1.99594607095361
C	4.15229106091881	3.44193188812993	-2.72297488451487
H	4.12237045385507	2.60500216338535	-3.42633551103180
H	3.49904813945089	4.22809180457675	-3.11187766586094
H	5.17319883767802	3.83114839615031	-2.70866991646406
O	0.14329962880640	1.06288004013454	1.47526026798201
C	0.42948544628451	1.47878864961076	0.15597394790269
H	0.42559841235740	0.56670438823377	-0.52855624949747
H	-0.28306180588351	2.22532500432940	-0.19644610662583
H	-0.81331886964345	1.00155037110369	1.58881627376423
C	1.85359196907301	2.00917426477286	0.09499582603572
H	1.91084423195913	2.85335343139745	0.78948560530765
H	2.53042375531715	1.23504343383950	0.46880200795113

$Mo(CO)_5$-1-pentanol (C2H-bound)

Mo	-1.23943165105557	-0.15702319885366	-1.90394303795108
C	0.25884204867765	-0.46477086557017	-3.27067655028898
C	-0.76026088162346	-1.92688505142394	-0.96599026309192
C	-2.43761920174142	-1.13752860497507	-3.08551386495192
C	-1.76814952873527	1.56407342441667	-2.88549010486313
C	-2.80065468843254	0.08589281014210	-0.58674410561588
O	1.04707588825249	-0.67418001357800	-4.07867587491081
O	-0.53213159938805	-2.93990479879528	-0.48057014621561
O	-3.15496793342111	-1.71435749679797	-3.79000273473808
O	-2.09753877660965	2.49300905479172	-3.47423130526232
O	-3.71042304946064	0.18442556857276	0.10404417285012
C	1.54531895697270	2.39709321882997	-0.42095267393930
H	2.38818016586611	2.40249777019013	0.27905245332515
C	2.08201338372701	2.38939815129527	-1.85144933867530
H	2.65353002564650	1.47000424249059	-2.01585867310548
H	1.24221598509559	2.36548718332296	-2.55384114644543
H	0.98321004950046	3.32010772376550	-0.24377129175899
C	2.96154845194278	3.60264337362709	-2.15342633703071
H	2.40030320981379	4.53283122672655	-2.02910686003425
H	3.82282833115644	3.63976675541350	-1.48061241863828
H	3.33703437604166	3.57146854875569	-3.17875118651260
O	-0.50851054629456	-0.01531455711107	1.62902089242178
C	0.21874213246482	1.18830796035304	1.37382277879728
H	-0.40229772737359	2.06521177761667	1.58350659409743
H	1.10933732727132	1.23656023365219	2.01161721485106
H	-0.93878365243879	0.06732467499938	2.48889234201316
C	0.66002012999302	1.19468849291713	-0.08028611020802
H	1.18150397878565	0.26257998695437	-0.30807860120285
H	-0.26961710163333	1.30098585527168	-0.70402056391490

$Mo(CO)_5$-1-pentanol (C3H-bound)

Mo	-1.01307657023815	-0.11952463878589	-1.94872611530390
C	0.71683425976149	-0.50434833541007	-2.98644308666089
C	-0.64677188907398	-1.75015798867511	-0.75684956398271
C	-1.93846332184259	-1.31013778462191	-3.18286165005867
C	-1.44026754552562	1.42929257329191	-3.22894564701333
C	-2.80659730646066	0.20843610270976	-0.99687952223598
O	1.64406212575569	-0.76360552241356	-3.60974454024878
O	-0.47038423748498	-2.70467211421028	-0.14575163872477
O	-2.47975690888549	-2.02411800014027	-3.91644896145828
O	-1.72215306117679	2.23945161777356	-3.99017569850260
O	-3.84051136917421	0.35299937583208	-0.52217249070030
C	0.29880988979947	1.77089107626211	-0.00400058409353
H	-0.61970026497808	2.32024398279127	-0.22493188798233
C	1.51782744374116	2.59263669640763	-0.43716041606651
H	1.59534681578915	3.43949334535623	0.25234606977499
H	2.42073793977579	1.99116753055529	-0.29484420956815
H	0.33077589577446	0.81047753628950	-0.59409696828039
C	1.45478768894992	3.11385027960510	-1.86913811186699
H	1.41686632941738	2.29487526116180	-2.59175716251600
H	0.57110727788770	3.73901292679179	-2.02059422854418

H	2.33678294496278	3.71600682095618	-2.09877521875884
O	-0.72522392526833	0.48202264018585	3.40017216196005
C	-0.92803844690395	0.78345172123945	2.01336076503615
H	-1.18027147123516	-0.13158446648534	1.46478386935402
H	-1.75861936434620	1.48933150546883	1.89542373690908
H	-1.54036949902718	0.09620863539416	3.74306795033953
C	0.35465484142462	1.39127679416433	1.47826562805761
H	0.57058420810694	2.29992418110665	2.04903904352792
H	1.18360516047483	0.69875615239878	1.65095746960793

$Mo(CO)_5$-1-pentanol (C4H-bound)

Mo	-1.04302067561560	-0.03972339134965	-1.95176964491324
C	0.55723038160745	-0.12754343865646	-3.23528674022648
C	-0.49243940963473	-1.85557741867189	-1.17084737638747
C	-2.11016127478136	-1.01932110063345	-3.25662988171621
C	-1.64252496101572	1.71237708388179	-2.84502953574876
C	-2.69642262376424	0.00138466778843	-0.72906495799232
O	1.41654919849259	-0.20785245710564	-3.99074447194038
O	-0.21967859585837	-2.90695647922191	-0.80168319614825
O	-2.74027649231111	-1.61017925273193	-4.02815283099552
O	-2.00594936388647	2.65109807266988	-3.39459116071447
O	-3.65251885472630	-0.00907521289009	-0.09598985999944
C	0.90213526980597	1.25186332375040	1.32471215795058
H	1.95482880964712	0.96532964550652	1.23048285829989
C	0.37097863123923	1.64303344898020	-0.05826378853879
H	0.48406602183045	0.71826083301691	-0.69285435884343
H	-0.67912571574870	1.93270603539362	0.02377903035531
H	0.87410087220034	2.14869626794466	1.95322755423528
C	1.17881752807658	2.76249773409666	-0.71231526421766
H	1.15706485504857	3.65120646056513	-0.07699855787641
H	2.22146837598560	2.46362316423681	-0.84151096435979
H	0.77757968506415	3.03367121930004	-1.68960645985590
O	-0.08841073155055	-1.30597191863686	3.90257756835837
C	0.67872859138482	-0.21778516172619	3.36731514098083
H	0.61684606005531	0.65147312810087	4.03286008691078
H	1.73336052374721	-0.50585918354928	3.28184910916901
H	0.27800185771650	-1.53279475111961	4.76566484135340
C	0.11924408014623	0.12705372858984	1.99848200862891
H	0.15030130532172	-0.77115582175845	1.37397634366147
H	-0.93231729547669	0.41289423622964	2.10302063357075

$Mo(CO)_5$-1-pentanol (C5H-bound)

Mo	-1.09746121224576	-0.08627217563994	-1.92753462302740
C	0.83577730513264	-0.38933184085578	-2.55539489534854
C	-0.85418836298618	-1.54564954826793	-0.49842369361783
C	-1.68779914301848	-1.46682116870894	-3.17456668465908
C	-1.36853071742766	1.29355134516618	-3.42319393397438
C	-3.05127310407794	0.17047946138773	-1.34005535690451
O	1.89655532669212	-0.59358688619421	-2.94046155144955
O	-0.73635044628703	-2.40492976550016	0.25191871140420
O	-2.03858304373786	-2.27669898448264	-3.92451438925330

O	-1.53776884208860	2.01442194333099	-4.29921651240434
O	-4.15537474711689	0.27546345223165	-1.04881108656157
C	0.88451705899785	2.62942341111311	2.06359006989868
H	0.23628763014505	3.51111532918144	2.12265473095513
C	0.98538727774935	2.17479559896691	0.60643688866552
H	1.47449788677639	2.95424743478192	0.01434366023350
H	1.61833025923806	1.28487458104215	0.54315547790324
H	0.40046204092152	1.84420043057938	2.65521167261268
C	-0.38651915688034	1.86967108841995	0.00324623446028
H	-0.88151984698765	1.05865460135584	0.54069576976226
H	-1.04888749668006	2.73616441684572	0.01733315170733
H	-0.22768260537766	1.64950192373944	-1.08383058437335
O	3.47257453895981	3.68696597486314	4.59456774728237
C	2.14801509033027	3.41217226197637	4.11304801692629
H	1.68072054014977	2.62990189112739	4.72332504998256
H	1.52723649128192	4.31353128695073	4.18336397434810
H	3.40158758420200	3.97645346527092	5.51205245074195
C	2.25105628477303	2.95828039767128	2.66845480673470
H	2.73444388039779	3.74684128332819	2.08233107916088
H	2.89937769816447	2.07713433131896	2.61927683879408

References

1 G. Smolentsev, A. A. Guda, M. Janousch, C. Frieh, G. Jud, F. Zamponi, M. Chavarot-Kerlidou, V. Artero, J. A. Van Bokhoven and M. Nachtegaal, *Faraday Discuss.*, 2014, **171**, 259–273.
2 R. M. Jay, A. Banerjee, T. Leitner, R.-P. Wang, J. Harich, R. Stefanuik, H. Wikmark, M. R. Coates, E. V. Beale, V. Kabanova, A. Kahraman, A. Wach, D. Ozerov, C. Arrell, P. J. M. Johnson, C. N. Borca, C. Cirelli, C. Bacellar, C. Milne, N. Huse, G. Smolentsev, T. Huthwelker, M. Odelius and P. Wernet, *Science (1979).*, 2023, **380**, 955–960.
3 M. Ekimova, F. Hoffmann, G. Bekçioğlu-Neff, A. Rafferty, O. Kornilov, E. T. J. Nibbering and D. Sebastiani, *J. Am. Chem. Soc.*, 2019, **141**, 14581–14592.
4 F. Neese, *WIREs Computational Molecular Science*, 2022, **12**, 1–15.
5 F. Neese, F. Wennmohs, A. Hansen and U. Becker, *Chem. Phys.*, 2009, **356**, 98–109.
6 M. Cossi, N. Rega, G. Scalmani and V. Barone, *Journal of Chemical Physics*, 2001, **114**, 5691–5701.
7 J. Tao, J. P. Perdew, V. N. Staroverov and G. E. Scuseria, *Phys. Rev. Lett.*, 2003, **91**, 146401.
8 F. Weigend and R. Ahlrichs, *Physical Chemistry Chemical Physics*, 2005, **7**, 3297–3305.
9 A. D. Becke, *J. Chem. Phys.*, 1993, **98**, 1372–1377.
10 C. Lee, W. Yang and R. G. Parr, *Phys. Rev. B*, 1988, **37**, 785–789.
11 E. van Lenthe, E. J. Baerends and J. G. Snijders, *J. Chem. Phys.*, 1993, **99**, 4597–4610.
12 J. D. Rolfes, F. Neese and D. A. Pantazis, *J. Comput. Chem.*, 2020, **41**, 1842–1849.
13 J. L. Campbell and T. Papp, *At. Data Nucl. Data Tables*, 2001, **77**, 1–56.
14 S. Dapprich and G. Frenking, *J. Phys. Chem.*, 1995, **99**, 9352–9362.
15 T. Lu and F. Chen, *J. Comput. Chem.*, 2012, **33**, 580–592.
